# Association between *UCP2* gene 3’UTR I/D and A55V polymorphisms and neural tube defects susceptibility: systematic review, meta-analysis, and trial sequential analysis

**DOI:** 10.3389/fneur.2024.1411184

**Published:** 2024-07-16

**Authors:** Haokun Tian, Zhen Guan, Shen Li, Jianhua Wang

**Affiliations:** ^1^Laboratory of Translational Medicine, Beijing Municipal Key Laboratory of Child Development and Nutriomics, Capital Institute of Pediatrics, Beijing, China; ^2^Graduate School of Peking Union Medical College, Chinese Academy of Medical Sciences, Beijing, China

**Keywords:** *UCP2*, polymorphism, neural tube defects, meta-analysis, trial sequential analysis

## Abstract

**Aim:**

Our study aimed to assess the association between *UCP2* gene 3’ untranslated region insertion/deletion (3’UTR I/D) and A55V (alanine/valine) polymorphisms and neural tube defects (NTDs) susceptibility.

**Materials and methods:**

According to pre-determined inclusion and exclusion criteria, the article search was conducted to search articles published before October 2023. Two authors independently screened the included articles and extracted their basic characteristics. After quality evaluation, the meta-analysis and trial sequential analysis (TSA) were conducted using RevMan 5.4, Stata/MP 17, and TSA 0.9.5.10 Beta. Subgroup analysis was conducted based on country and case group composition. Sensitivity analysis was conducted using a one-by-one exclusion method. Begg’s and Egger’s tests were used to evaluate publication bias.

**Results:**

A total of seven articles were included. Overall meta-analysis revealed significant heterogeneity among the included studies for 3’UTR I/D polymorphism of the *UCP2* gene. Significant statistical data indicated that those with the DD genotype and D allele had higher chances of NTD compared to those with the II genotype and the I allele, respectively. The combined result of II vs. ID was not statistically significant. A55V variation showed no statistical significance in the risk of NTD, despite the absence of significant heterogeneity across the included studies. Most of the heterogeneity was resolved after subgrouping, and a higher risk of the ID genotype was found than the II genotype for Chinese people. Genotyping NTD patients or their mothers was not a factor affecting the heterogeneity. Sensitivity analysis and publication bias analysis suggested that positive findings supported our results.

**Conclusion:**

The *UCP2* gene 3’UTR I/D polymorphism increased the likelihood of developing NTDs in the Chinese population, with the D allele being the risk factor, which contributed to the understanding of the genetic basis of NTDs. TSA indicated that more high-quality original studies were needed in the future for further validation.

## 1 Introduction

Neural tube defects (NTDs) are a class of congenital abnormalities distinguished by incomplete closure of the neural tube throughout the process of embryonic development (1). These abnormalities pose a serious global public health concern as they can result in serious neurological complications. NTDs have a complex etiology that includes environmental, dietary, and genetic factors (2, 3). Figuring out the genetic mechanisms that contribute to NTD susceptibility is essential for achieving a comprehensive understanding of their pathogenesis and creating effective prevention and treatment measures.

Gene polymorphisms, which represent specific variations in DNA sequence, are essential for modifying susceptibility to various diseases and conditions. These variations alter the expression and function of genes, contributing to the complex genetic mechanism underlying NTDs. For example, it has been confirmed that specific gene polymorphisms affect the susceptibility to NTDs (4, 5). As a member of the mitochondrial anion carrier protein family, uncoupling protein 2 (*UCP2*) is primarily responsible for regulating the reactive oxygen species that are produced by the mitochondria (6, 7). The *UCP2* gene, which is located on chromosome 11q13, has been considered a potential candidate in the genetic landscape of NTDs (8, 9). *UCP2* variants may play an important role in energy metabolism, weight regulation, and preventing the accumulation of reactive oxygen species. These conditions, primarily through obesity and diabetes, are considered risk factors for NTDs (10, 11). Numerous physiological processes, such as the regulation of oxidative stress and mitochondrial function, have been linked to the 3′ untranslated region (3’UTR) insertion/deletion (I/D) and A55V polymorphisms within the *UCP2* gene (12–14). Many studies have been conducted to assess the link between these polymorphisms and the likelihood of suffering NTDs, but conflicting findings have left conclusions uncertain (10, 11, 15–19).

In order to provide a more robust and reliable estimate of the genetic contribution of *UCP2* to NTD susceptibility by pooling data from relevant studies, we conducted a thorough meta-analysis by systematically evaluating the relationship between the polymorphisms of 3’UTR I/D and A55V in the *UCP2* gene and the risk of NTDs. Additionally, to enhance the validity of our findings and control for potential false-positive results, trial sequential analysis (TSA) was performed as a statistical method designed to assess the cumulative evidence and determine whether further studies were warranted (20). In this way, our findings aimed to provide robust insights into the genetic basis of NTDs, guide future research, and influence clinical strategies for the prevention and management of these congenital anomalies.

## 2 Materials and methods

### 2.1 Article inclusion and exclusion criteria

Our meta-analysis was performed based on the preferred reporting items for systematic reviews and meta-analyses (PRISMA) guideline (21). PROSPERO confirmed our meta-analysis registration under the ID CRD42023483551. The articles we included needed to be publicly published studies that investigated the connection between *UCP2* gene polymorphism and NTD susceptibility. The research object of the original study needed to be humans, regardless of age, gender, country, or race. The original study needed to have a clear control group and case group, with the number of people in each group and genotype provided. Articles with unrelated topics would be excluded. If the data were incomplete and could not be resolved after contacting the corresponding author via email, it would also be excluded.

### 2.2 Article searching strategy

Article searching was conducted using Web of Science, PubMed, Embase, CNKI (Chinese), Wangfang Data (Chinese), and VIP (Chinese) databases. Two authors independently searched articles published from the establishment of the database to October 2023 and cross-checked the results. If there were different results, it would be consulted with third-party experts for advice. The search terms included *UCP2*, uncoupling protein 2, NTDs, NTDs, spina bifida, anencephaly, encephalocele, spinal dysraphism, hydranencephaly, iniencephaly, myelomeningocele, meningocele, schizencephaly, lipomeningocele, and craniorachischisis. Table 1 shows the search strategy using PubMed as an example.

**Table 1:** The searching strategy of PubMed.

#1	*UCP2*
#2	Uncoupling protein 2
#3	#1 OR #2
#4	Neural tube defects
#5	NTDs
#6	Spina bifida
#7	Anencephaly
#8	Encephalocele
#9	Spinal dysraphism
#10	Hydranencephaly
#11	Iniencephaly
#12	Myelomeningocele
#13	Meningocele
#14	Schizencephaly
#15	Lipomeningocele
#16	Craniorachischisis
#17	#4 OR #5 OR #6 OR #7 OR #8 OR #9 OR #10 OR #11 OR #12 OR #13 OR #14 OR #15 OR #16
#18	#3 AND #17

### 2.3 Article screening and data extraction

Two authors independently conducted article screening and cross checked the results. Any different results would be resolved with the advice of third-party experts. Firstly, for articles that were repeatedly retrieved in multiple databases, only one of the records would be retained. Read the title and abstract of the articles firstly for preliminary screening, and then excluded those that obviously did not match the inclusion criteria. After then, read the full content for final screening and recorded the reasons for each piece that was excluded. Two authors independently extracted data for each original study, including the name of first author, publication year, country, the type of case group, and the number of people in each group and genotype.

### 2.4 Article quality evaluation

Using the Newcastle–Ottawa scale, two authors independently assessed and cross-checked the quality of original studies. If there were different results, it would be consulted with third-party experts for advice. Articles evaluated as low quality would be excluded before being included in the meta-analysis.

### 2.5 Statistical method

Meta-analysis and TSA were conducted using Stata MP 17 (StataCorp LLC, Texas, United States), Review Manager 5.4.1 (The Cochrane Collaboration, London, United Kingdom), and TSA 0.9.5.10 Beta (Copenhagen Trial Unit, Copenhagen, Denmark) (α = 0.05). If the *p*-value of the heterogeneity test was greater than 0.1, it indicated that there was no significant heterogeneity, and a fixed effect model would be used for meta-analysis. If *p* < 0.1 was found, significant heterogeneity was indicated. The random-effects model will be used to attempt to explain and handle the heterogeneity through subgroup analysis. Combined odds ratios (*ORs*), 95% confidence intervals (*CIs*), and *p*-values were recorded. A strategy of one-by-one exclusion was used to conduct sensitivity analysis. To assess publication bias, Begg’s and Egger’s tests were applied. TSA was applied to evaluate whether the number of samples incorporated into the meta-analysis had reached the “required information size,” and whether sufficient samples had been included to provide a “definitive” conclusion. This method was particularly suited for our analysis as it provided a suggested endpoint for clinical original studies on this topic, ensuring that the conclusions drawn were robust and reliable. Additionally, for meta-analysis that included a limited number of studies, it indicated whether further research was needed to validate the conclusions (20).

## 3 Results

### 3.1 Article searching, screening, data extraction, and quality evaluation results

**Figure 1:**
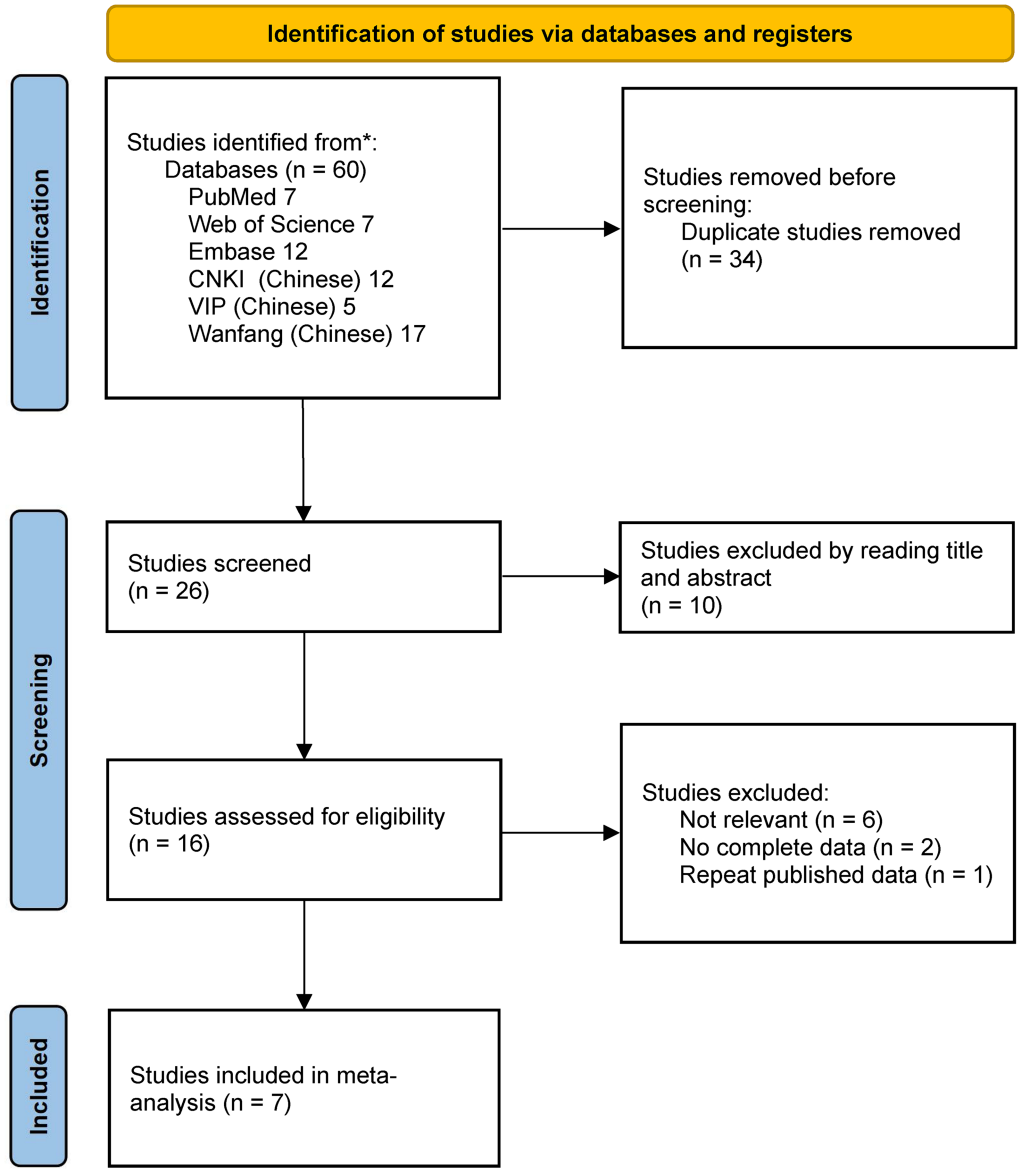
Flow diagram of searching and screening.

A total of 60 records were retrieved through the article searching process. After the step-by-step screening, a total of seven articles met the final inclusion criteria (10, 11, 15–19). Among them, there were five studies in China and two studies in the United States of America (United States). For 3’UTR I/D, the original studies had a total of 1848 samples, including 864 in the case group and 984 in the control group. For A55V, the original studies had a total of 1,572 samples, including 781 in the case group and 791 in the control group. The flow diagram of article searching and screening is shown in Figure 1.

The result of the article quality evaluation indicated that the quality of all the articles mentioned above met the inclusion criteria for meta-analysis. The results of data extraction are shown in Tables 2, 3. For the study conducted by Mitchell A et al., the case groups of children and mothers shared the same control group. As the shared control group could not be reused, the sample size of each genotype in the control group would be halved when the case groups of children and mothers were included in the same meta-analysis (22).

**Table 2:** Basic characteristics of original studies on the *UCP2* gene 3’UTR I/D polymorphism.

First author	Year	Country	Case group type	Case			Control		
				II	ID	DD	II	ID	DD
Volcik KA	2003	USA	Children	0	9	15	5	8	7
Zhang T	2006	China	Mother	1	13	102	8	28	80
Mitchell A a	2009	USA	Children	10	64	85	13	135	177
Mitchell A b	2009	USA	Mother	12	60	81	13	135	177
Liu ZZ	2010	China	Mother	10	30	76	29	45	42
Wang JH	2011	China	Mother	3	36	101	5	48	198
Liu ZZ	2012	China	Mother	5	18	133	12	46	98

I, insertion; D, deletion.

**Table 3:** Basic characteristics of original studies on the *UCP2* gene A55V polymorphism.

First author	Year	Country	Case group type	Case			Control		
				AA	AV	VV	AA	AV	VV
Volcik KA	2003	USA	Children	0	2	21	1	2	16
Zhang T	2006	China	Mother	39	77	0	29	87	0
Mitchell A a	2009	USA	Children	52	82	22	110	169	42
Mitchell A b	2009	USA	Mother	50	77	24	110	169	42
Liu ZZ	2010	China	Mother	39	75	2	38	70	8
Liu ZZ	2012	China	Mother	60	86	10	54	85	17
He JF	2013	China	Mother	27	28	8	32	24	7

A, alanine; V, valine.

### 3.2 Overall meta-analysis results

By incorporating data from all individuals, the overall meta-analysis of the population was conducted, with the results represented in Table 4.

**Table 4:** Overall meta-analysis results of the *UCP2* gene 3’UTR I/D and A55V polymorphisms.

	Polymorphism	Heterogeneity *p*	*OR*	*p*	95% *CI*
3’UTR I/D	II vs. ID	0.0004^*^	0.48	0.14	[0.18, 1.27]
	II vs. DD	<0.00001^*^	0.57	0.048^*^	[0.33, 0.995]
	I vs. D	<0.00001^*^	0.58	0.04^*^	[0.35, 0.98]
A55V	AA vs. AV	0.27	1.08	0.70	[0.74, 1.57]
	AA vs. VV	0.28	1.07	0.69	[0.76, 1.52]
	A vs. V	0.62	1.03	0.65	[0.89, 1.20]

For heterogeneity *Q*-test, ^*^ represents *p* < 0.10. For meta-analysis and subgroup analysis, ^*^ represents *p* < 0.05. OR, odds ratio; CI, confidence interval; UTR, untranslated region; I: insertion; D, deletion; A, alanine; V, valine.

**Figure 2:**
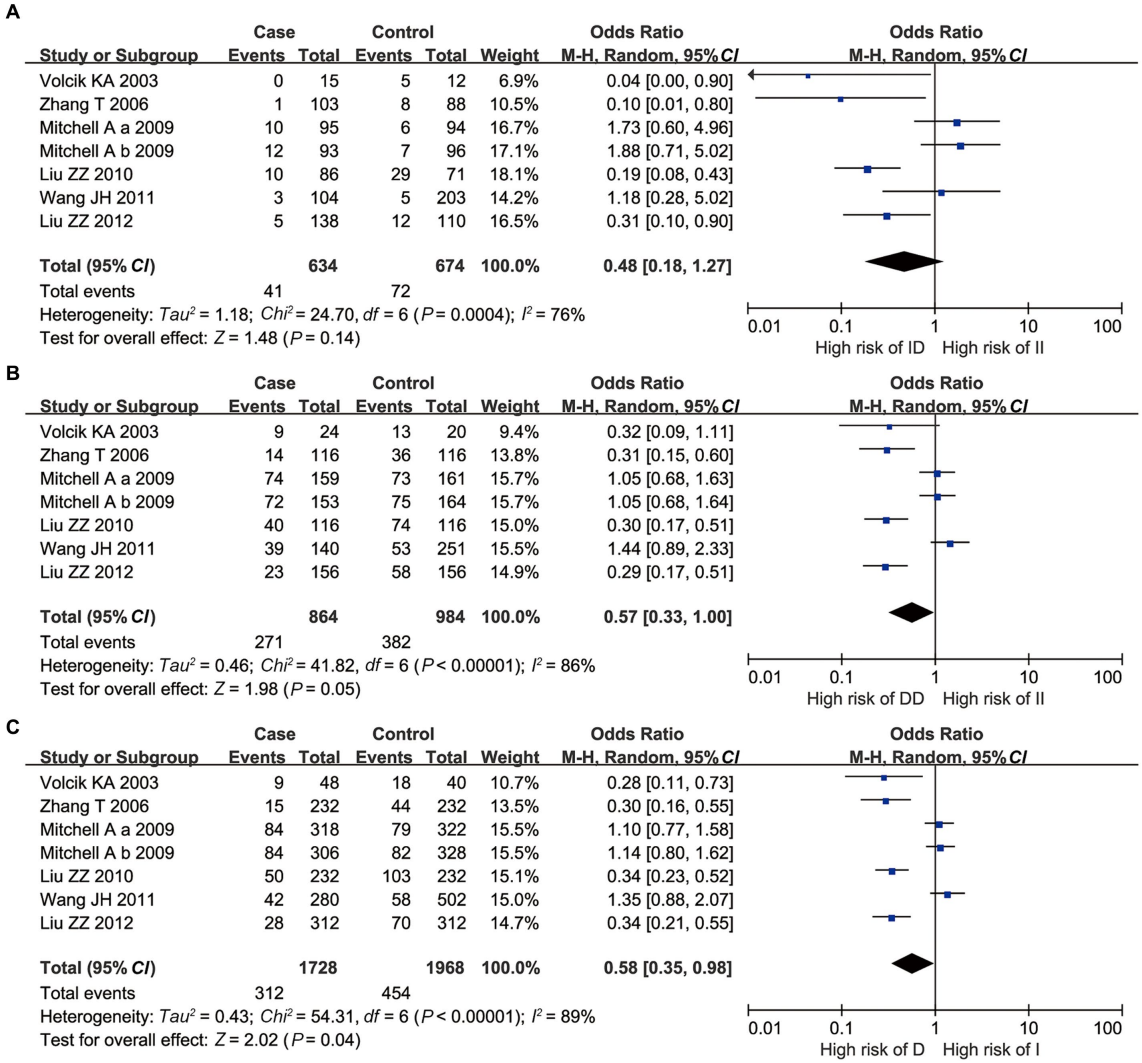
Overall meta-analysis results of the 3’UTR I/D polymorphism of *UCP2* gene. **(A)** II vs. ID; **(B)** II vs. DD; and **(C)** I vs. D.

For 3’UTR I/D, all II vs. ID, II vs. DD, and I vs. D polymorphisms had a significant *p*-value of heterogeneity in the *Q*-test, indicating that high heterogeneity existed. Therefore, the random-effects model was applied. II vs. ID polymorphism showed that the *OR* = 0.48, with *p* = 0.14 and 95% *CI* [0.18, 1.27], indicating no statistical significance (Figure 2A). However, for II vs. DD and I vs. D polymorphisms, the combined *ORs* were 0.57 and 0.58, with 95% *CI* [0.33, 0.995] and [0.35, 0.98], respectively. This showed that individuals with the DD genotype would suffer a higher risk of NTDs than those with the II genotype, and individuals with the D allele would suffer a higher risk of NTDs than those with the I allele, with significant statistical evidence (Figures 2B,C).

**Figure 3:**
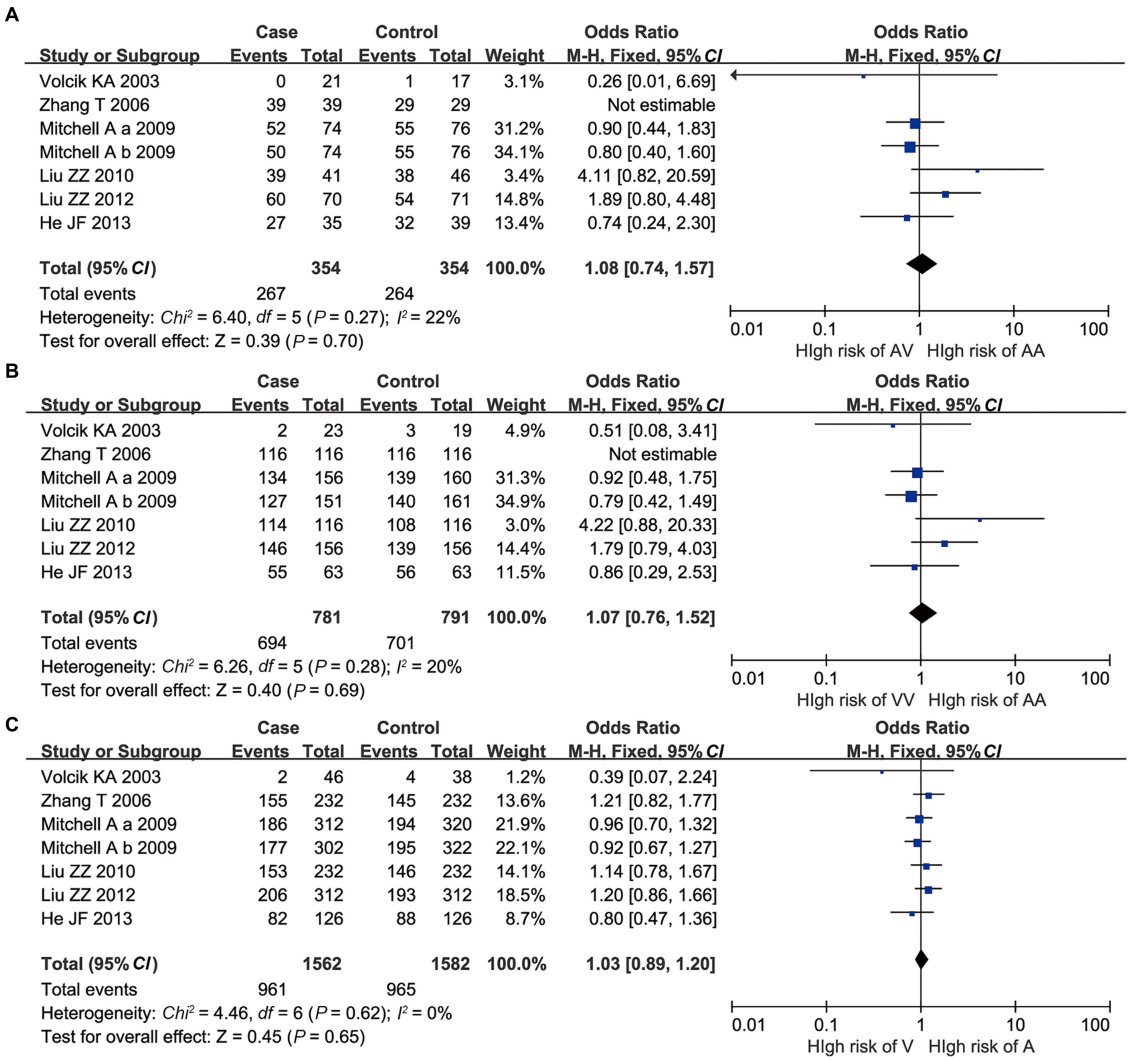
Overall meta-analysis results of A55V polymorphism of *UCP2* gene. **(A)** AA vs. AV; **(B)** AA vs. VV; and **(C)** A vs. V.

For A55V, all AA vs. AV, AA vs. VV, and A vs. V polymorphisms had no significant *p*-value of heterogeneity in the *Q*-test, indicating that low heterogeneity existed. Besides, all the *p-values* of the overall meta-analysis showed that no statistical significance was found under the fixed effect model (*p* > 0.05, Figure 3).

### 3.3 Subgroup analysis results based on country

Subgroup analysis was carried out based on country (Table 5). For 3’UTR I/D II vs. ID polymorphism, the heterogeneity was resolved after subgrouping, with a heterogeneity *Q*-test *p*-value of 0.13 > 0.1 in the Chinese subgroup. This situation was similar in II vs. DD polymorphism, with a heterogeneity *Q*-test *p*-value of 0.19 > 0.1 in the United States subgroup. For the Chinese population, the results of the II vs. ID meta-analysis showed *OR* = 0.27 and *p* < 0.0001, with 95% *CI* [0.15, 0.46], indicating that individuals carrying ID genotype had a higher risk of NTDs than those carrying II genotype, and the results were statistically significant. However, for the USA population, there was no statistical evidence to suggest an association between the risk of NTDs considering all II vs. ID, II vs. DD, and I vs. D polymorphisms (*p* > 0.05). These findings were different from the results of the overall meta-analysis.

**Table 5:** Subgroup analysis results based on the country of the *UCP2* gene 3’UTR I/D and A55V polymorphisms.

	Polymorphism	China				USA			
		Heterogeneity *p*	*OR*	*p*	95% *CI*	Heterogeneity *p*	*OR*	*p*	95% *CI*
3’UTR I/D	II vs. ID	0.13	0.27	<0.00001^*^	[0.15, 0.46]	0.05^*^	1.03	0.97	[0.26, 4.08]
	II vs. DD	<0.00001^*^	0.45	0.06	[1.09, 1.05]	0.19	0.98	0.88	[0.72, 1.32]
	I vs. D	<0.00001^*^	0.47	0.04^*^	[0.23, 0.98]	0.02^*^	0.86	0.58	[0.50, 1.47]
A55V	AA vs. AV	0.20	0.64	0.11	[0.89, 3.02]	0.75	0.82	0.43	[0.50, 1.34]
	AA vs. VV	0.24	1.67	0.08	[0.93, 3.00]	0.83	0.83	0.40	[0.54, 1.29]
	A vs. V	0.61	1.12	0.24	[0.93, 1.36]	0.61	0.93	0.50	[0.74, 1.16]

For heterogeneity *Q*-test, ^*^ represents *p* < 0.10. For meta-analysis and subgroup analysis, ^*^ represents *p* < 0.05. OR, odds ratio; CI, confidence interval; UTR, untranslated region; I, insertion; D, deletion; A, alanine; V, valine.

For A55V polymorphism, the findings after subgrouping were similar to overall meta-analysis results, indicating that no significant statistical evidence was found to support the association with NTD risk.

### 3.4 Subgroup analysis results based on case group composition

Subgroup analysis was carried out based on case group composition (Table 6). For both 3’UTR I/D and A55V polymorphisms, the heterogeneity seemed to have no significant change after subgrouping according to the case group composition (mother or children). The situations had not changed, presenting as *p* > 0.1 for II vs. ID, II vs. DD, I vs. D of 3’UTR I/D polymorphism, and AA vs. AV, AA vs. VV, and A vs. V of A55V polymorphism. This indicated that genotyping NTD patients or their mothers was not a factor affecting the heterogeneity of the meta-analysis. Besides, for 3’UTR I/D, significant II vs. DD and I vs. D polymorphisms in the overall meta-analysis did not show significant statistical differences after subgrouping based on case group composition.

**Table 6:** Subgroup analysis results based on the case group composition of *UCP2* gene 3’UTR I/D and A55V polymorphisms.

	Polymorphism	Mother				Children			
		Heterogeneity *p*	*OR*	*p*	95% *CI*	Heterogeneity *p*	*OR*	*p*	95% *CI*
3’UTR I/D	II vs. ID	0.003^*^	0.46	0.19	[0.15, 1.46]	0.02^*^	0.35	0.57	[0.01, 13.12]
	II vs. DD	<0.00001^*^	0.54	0.09	[0.27, 1.09]	0.08^*^	0.68	0.49	[0.22, 2.05]
	I vs. D	<0.00001^*^	0.57	0.09	[0.29, 1.09]	0.008^*^	0.60	0.45	[0.16, 2.24]
A55V	AA vs. AV	0.13	1.14	0.54	[0.75, 1.75]	0.46	0.86	0.61	[0.47, 1.56]
	AA vs. VV	0.12	1.13	0.53	[0.76, 1.69]	0.56	0.87	0.62	[0.51, 1.49]
	A vs. V	0.53	1.05	0.52	[0.90, 1.23]	0.32	0.94	0.64	[0.71, 1.23]

For heterogeneity *Q*-test, ^*^ represents *p* < 0.10. For meta-analysis and subgroup analysis, ^*^ represents *p* < 0.05. OR, odds ratio; CI, confidence interval; UTR, untranslated region; I, insertion; D, deletion; A, alanine; V, valine.

### 3.5 Sensitivity analysis results

The one-by-one exclusion method was used for sensitivity analysis. For 3’UTR I/D polymorphism, taking II vs. ID as an example, the combined *OR* remained less than 1 and *p* remained higher than 0.05 after excluding any single original studies. It indicated that the meta-analysis results were relatively stable and less affected by changes in a single study. The situation remained the same for A55V polymorphism, taking AA vs. AV as an example.

### 3.6 Publication bias analysis results

**Figure 4:**
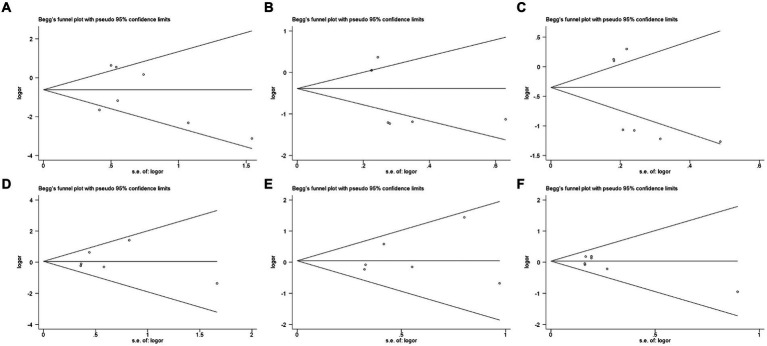
Funnel plots of publication bias analysis. **(A)** II vs. ID; **(B)** II vs. DD; **(C)** I vs. D; **(D)** AA vs. AV; **(E)** AA vs. VV; and **(F)** A vs. V.

According to Begg’s test, the qualitative funnel plot is shown in meta-analysis were reliab Figure 4. All the included studies presented in the plot, and distributed symmetrically along the axis, indicating low risk of publication bias. The quantitative data were calculated to reach more accurate conclusions, with all the *p* > 0.05, and the quantitative Egger’s test of all the polymorphisms showed *p* > 0.05 as well. All of the above indicated no significant publication bias, and the results of the meta-analysis were reliable. All the quantitative data are presented in Table 7.

**Table 7:** Publication bias analysis results based on Begg’s and Egger’s test.

	Polymorphism	Begg’s test		Egger’s test		
		*Z*	*p*	*t*	*p*	95% *CI*
3’UTR I/D	II vs. ID	0.90	0.368	−0.52	0.627	[−7.305, 4.859]
	II vs. DD	0.60	0.548	−1.50	0.194	[−14.271, 3.760]
	I vs. D	0.30	0.764	−1.61	0.168	[−15.888, 3.642]
A55V	AA vs. AV	0.38	0.707	0.23	0.831	[−3.345, 3.941]
	AA vs. VV	0.75	0.452	0.65	0.554	[−2.883, 4.628]
	A vs. V	0.90	0.368	−1.50	0.194	[−3.611, 0.950]

For publication bias analysis, ^*^ represents *p* < 0.05. CI, confidence interval; UTR, untranslated region; I, insertion; D, deletion; A, alanine; V, valine.

### 3.7 Trial sequential analysis results

**Figure 5:**
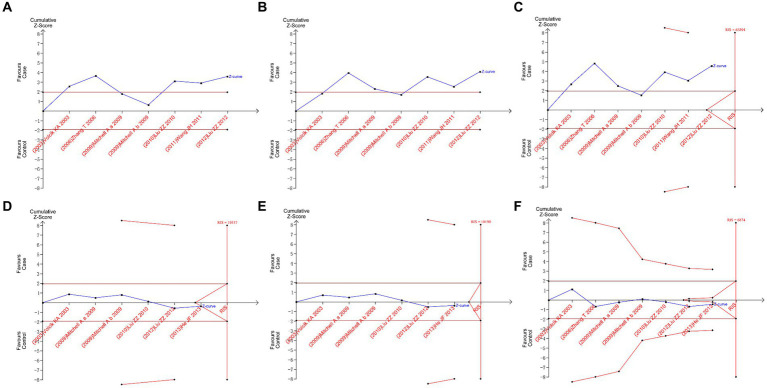
Trial sequential analysis plots. **(A)** II vs. ID; **(B)** II vs. DD; **(C)** I vs. D; **(D)** AA vs. AV; **(E)** AA vs. VV; and **(F)** A vs. V.

The boundary type of hypothesis testing was set as two-sided tests, and Type I error was defined as 5%. For the required information size (RIS), statistical power was defined as 80%. Based on clinical experience, the relative risk reduction rate was defined as 35%, and the event rate in the control group was defined as 3%. The results of TSA are shown in Figure 5. All plots showed that the Z-curve neither intersects the threshold line nor reaches RIS. This showed that we did not have sufficient confidence that the results of the meta-analysis did not need to be verified by subsequent original studies; that is, the minimum amount of sample required to draw a “definitive” conclusion had not yet been reached, suggesting the necessity for further original studies to validate the conclusion of this meta-analysis in the future (20).

## 4 Discussion

Prior research has indicated associations between *UCP2* gene polymorphisms, specifically 3’UTR I/D and A55V, and diverse health conditions, with their conflicts prompting our exploration of their potential correlation. The involvement of *UCP2* in mitochondrial function influencing energy metabolism was reported to be crucial for neural tube development. The meta-analysis conducted in this study aimed to explore the association between *UCP2* gene polymorphisms (3’UTR I/D and A55V) and the susceptibility to NTDs. Our findings would reveal several key insights that warranted discussion.

The overall meta-analysis results for the 3’UTR I/D polymorphism indicated a significant association with NTD risk in the II vs. DD and I vs. D comparisons, whereas the II vs. ID polymorphism showed no statistical significance. These results suggested that individuals with the DD genotype might face a higher risk of NTDs than those with the II genotype, and individuals with the D allele might be at an elevated risk compared to those with the I allele. However, a lack of significance in the II vs. ID comparison was found. Subgroup analysis based on the country of origin revealed divergent outcomes. The II vs. ID meta-analysis demonstrated a significant association with an increased NTD risk in the Chinese population, while no such association was observed in the overall United States populations. These differences could be attributed to various factors, including genetic diversity, environmental influences, and dietary habits. For example, variations in folate metabolism, which is crucial for neural tube closure, might differ between populations. Additionally, differences in sample sizes and study designs could contribute to these discrepancies. Future research should focus on exploring these population-specific factors in greater detail to better understand the genetic and environmental interactions affecting NTD risk. Identifying these differences was crucial for developing targeted prevention strategies and improving public health outcomes. Subgroup analysis based on case group composition did not yield significant changes in heterogeneity, suggesting that genotyping NTD patients or their mothers did not significantly impact the overall meta-analysis results.

The results of the A55V polymorphism meta-analysis indicated no significant association with NTD risk, and this lack of significance persisted in subgroup analyses based on country and case group composition. These findings suggested that the A55V polymorphism might not play a substantial role in NTD susceptibility, at least in the populations considered in the included studies.

Furthermore, the sensitivity analysis demonstrated the stability of our findings, as the exclusion of any single study did not substantially alter the outcomes. Regarding publication bias, it was generally considered that a symmetrical funnel plot from Begg’s test indicated no publication bias. Given the small sample size in this study, we also considered the quantitative Begg’s test, where *p* > 0.05 indicated no significant publication bias. Similarly, in Egger’s test, all groups had *p*-values greater than 0.05. These pieces of evidence suggested that the reliability of the conclusions in this meta-analysis was minimally affected by publication bias from a statistical standpoint. This enhanced the credibility of our meta-analysis results, suggesting that the conclusions drawn were reliable.

The impact of *UCP2* gene polymorphisms on NTDs prompted exploration into potential mechanisms. While the exact functional implications remained unclear, *UCP2*, involved in mitochondrial regulation, might affect bioenergetics and redox balance through specific polymorphisms, influencing NTD development (12–14, 23). Polymorphisms might change nutrient availability and interfere with vital pathways in neural tube closure because of the role of *UCP2* in energy homeostasis (24–28). Furthermore, the connection to immunological responses and inflammation, possible interactions with environmental factors, and impact on epigenetic modifications also highlighted how *UCP2* was involved in the risk of disease (29–31). Recognizing these mechanisms was essential in formulating interventions for the prevention and management of NTDs. Multiple studies have confirmed that obesity was an independent risk factor for NTDs during pregnancy, with a risk increase of 2 to 5 times (10). Additionally, women with diabetes and obesity experience numerous metabolic abnormalities, such as hyperglycemia, which are associated with a higher risk of birth defects (11). Since *UCP2* variants might play an important role in energy metabolism, weight regulation, and preventing the accumulation of reactive oxygen species, these conditions were also considered NTD risk factors through obesity and diabetes (10, 11). Therefore, variations in *UCP2* were attractive candidates for screening potential risk factors for NTDs.

It was crucial to recognize some limitations. First of all, differences in study designs, population characteristics, and genotyping techniques could have a significant impact on the heterogeneity seen in certain analyses. Although subgroup analysis had addressed some heterogeneity issues, there were still issues in certain analyses that might affect the reliability of the summary results. Furthermore, even though the analysis of publication bias suggested a low risk of bias, it was not completely possible to rule out the possibility of unpublished or undiscovered studies. Finally, a limited number of studies were included in this meta-analysis, which was also indicated by TSA, thus resulting in the limited number of included countries, which restricted the applicability of the study results to other populations and ethnic groups (20). Nevertheless, the study of *UCP2* gene polymorphisms in NTDs was still a novel, contentious, and stimulating field of research that could provide foundational knowledge for further research in this developing field.

In conclusion, our meta-analysis sheds significant information on the possible relationship between NTD susceptibility and *UCP2* gene polymorphisms. The *UCP2* gene 3’UTR I/D polymorphism increased the likelihood of developing NTDs, with D being the risk factor, which was mainly seen in the Chinese population. To confirm and expand on our findings and ultimately provide an improved understanding of the genetic basis of NTDs, more well-designed original studies are expected to be carried out in the future.

## Data availability statement

The original contributions presented in the study are included in the article/supplementary material, further inquiries can be directed to the corresponding author.

## Author contributions

HT: Writing – original draft, Writing – review & editing. ZG: Writing – original draft, Writing – review & editing. SL: Writing – original draft, Writing – review & editing. JW: Writing – original draft, Writing – review & editing.
